# Supplementary material for: Sulfane Sulfur Regulates LasR-Mediated Quorum Sensing and Virulence in Pseudomonas aeruginosa PAO1
Source: Antioxidants (Basel). 2021 Sep 21;10(9):1498. doi: 10.3390/antiox10091498 (PMC8469610; doi:10.3390/antiox10091498)
Supplement: Supplementary file 1 [file antioxidants-10-01498-s001.zip › antioxidants-1353323-supplementary.pdf]

# Supplementary Materials

**Sulfane sulfur regulates LasR-mediated quorum sensing and virulence in *Pseudomonas aeruginosa* PAO1**

Guanhua Xuan[1#], Chuanjuan Lü[1#], Huangwei Xu[1], Kai Li[1], Huaiwei Liu[1], Yongzhen Xia[1*], Luying Xun[1,2*]

**Affiliations:**

[1]State Key Laboratory of Microbial Technology, Shandong University, Qingdao, 266237, People's Republic of China.

[2]School of Molecular Biosciences, Washington State University, Pullman, WA, 99164-7520, USA.

[#] The authors contributed equally. LC started the research; GX completed the research.

* Corresponding authors:

Yongzhen Xia: xiayongzhen2002@email.sdu.edu.cn; Tel. +86 532 58631572.

Luying Xun: luying_xun@vetmed.wsu.edu; Tel. +1-509-335-2787.

**Contents:**
**Figure S1 —Figure S6**
**Table S1—Table S5**

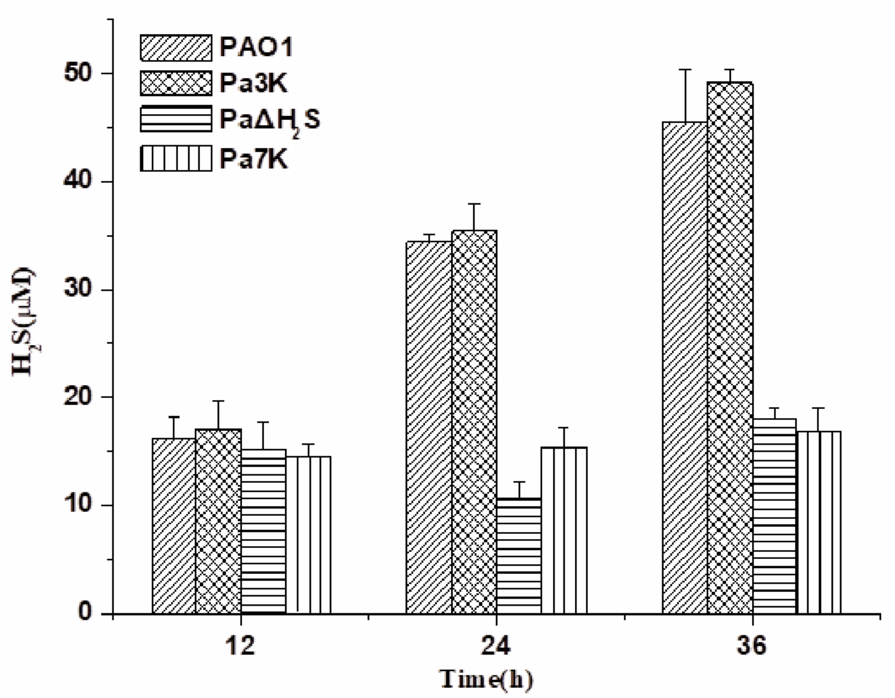


**Figure S1. The production of $H_2S$ in PAO1 and its mutants.** Overnight cultures were inoculated into LB medium at an initial $OD_{600}$ of 0.05. The production of $H_2S$ was assessed at 12 h, 24 h and 36 h with the mBBr method. All data are averages of three samples with standard deviations (error bars).

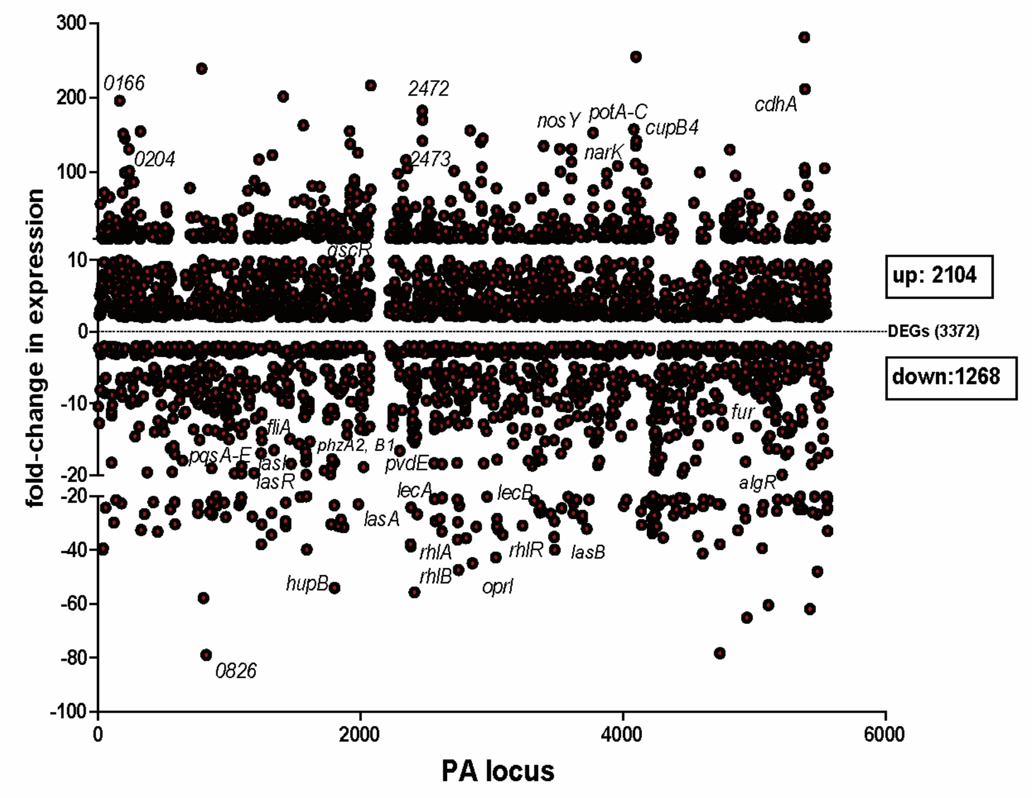


**Figure S2. Transcriptomic analysis of PAO1 and the PaΔ$H_2$S mutant.** Data are expressed relative to the wild-type strain results. The fold change in expression for each locus tag is indicated. The locus identifier PA_abcd, where "abcd" represents the locus number. The most representative downregulated genes: PA4944, RNA-binding protein Hfq; PA1804, DNA-binding protein HU; PA2853, outer membrane lipoprotein OprI; PA3479, rhamnosyltransferase subunit A; PA2741, 50S ribosomal protein L20; PA3477, transcriptional regulator RhlR. The most representative upregulated genes: PA5386, 3-hydroxybutyryl-CoA dehydrogenase; PA0166, transporter; PA2472, major facilitator superfamily transporter; PA4083, chaperone CupB4; PA2836, secretion protein; PA0324, ABC transporter permease.

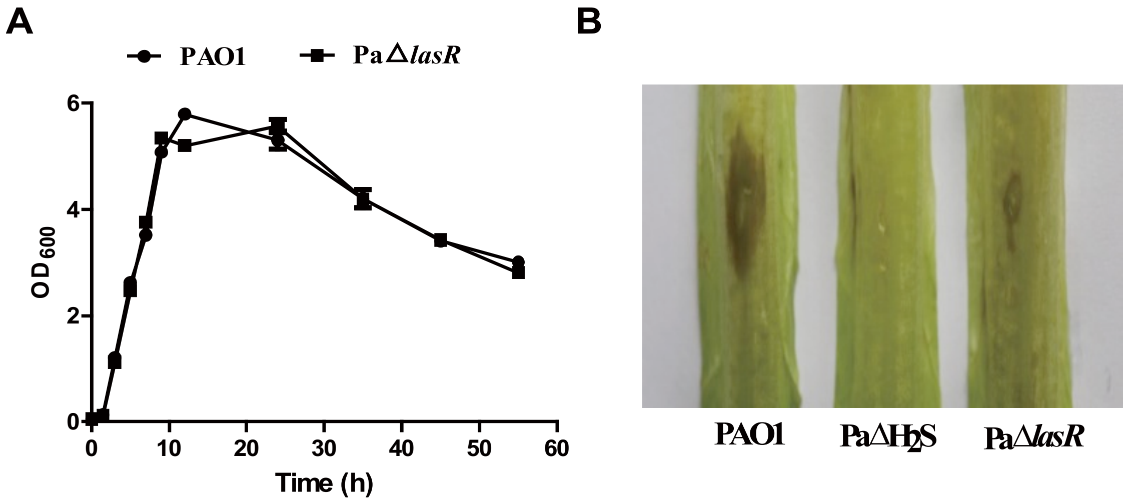


**Figure S3. The effect of *lasR* deletion in *P. aeruginosa* PAO1 on the growth and pathogenicity. (A)** Growth of *P. aeruginosa* PAO1 and its mutant in LB medium at 37$^{o}$C. Overnight cultures were inoculated into LB medium at an initial $OD_{600}$ of 0.05. Data are averages of three samples with standard deviations (error bars). **(B)** Virulence of PAO1 and its mutant strains on lettuce.

**A** **(Peptide 1a)**

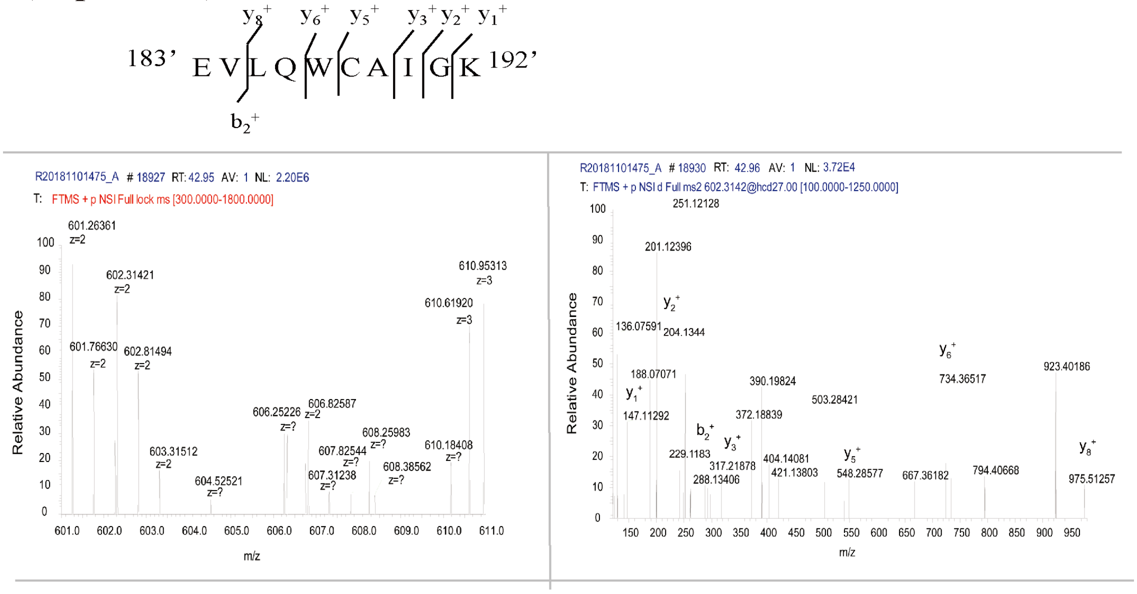


Sequence: EVLQWCAIGK, C6-Carbamidomethyl (57.02146 Da) Charge: +2, Monoisotopic m/z: 602.31421 Da (+1.01 mmu/+1.68 ppm), MH+: 1203.62114 Da.

**B** **(Peptide 1b)**

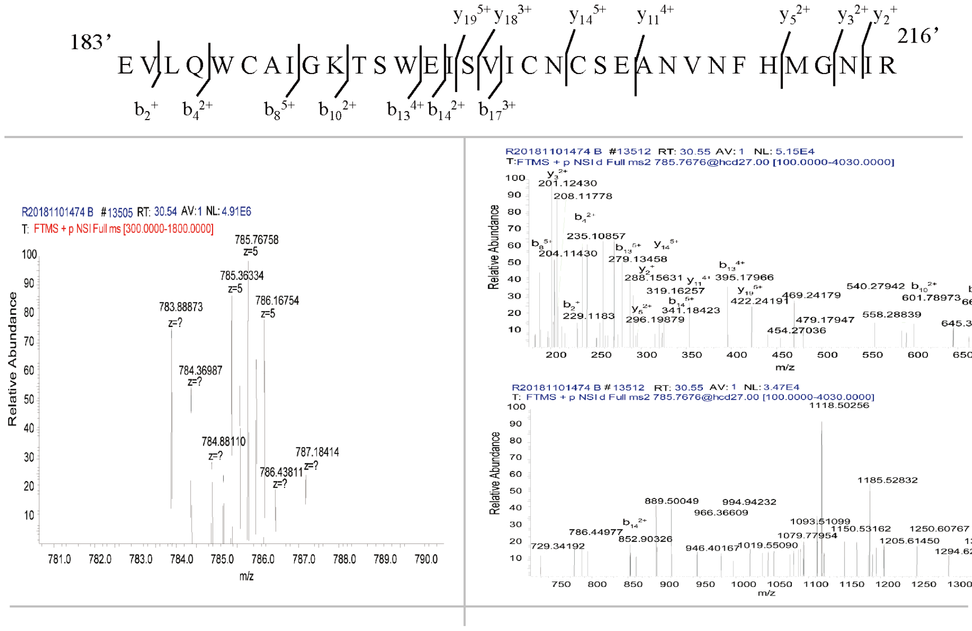


Sequence:EVLQWCAIGKTSWEISVICNCSEANVNFHMGNIR, C6-S-O-Carbamidomethyl (73.02000 Da), Charge: +5, Monoisotopic m/z: 785.76758 Da (-4.77 mmu/-6.08 ppm), MH+:3924.80878 Da.

**Figure S4. LTQ-Orbitrap tandem mass spectrometry analysis of LasR (Peptides 1a&1b).**

**A) Peptide 1a in purified LasR and DTT-treated LasR.** A Graphical fragment map correlating the relevant peptide sequence with the observed fragmentation ions of Peptide 1a. Left, the 2+ charge state (m/z 602.31421) corresponds to a peptide containing $Cys^{188}$

modified by IAM (theoretical molecular mass, 1203.61909). Right, MS/MS fragmentation of the fragment (m/z 602.31421), in which the $y_1^+$ peak represents the last amino acid residue (K) of Peptide 1a and the $b_2^+$ peak represents the first two amino acid residues (EV) of Peptide 1a. Other peaks follow the same pattern.

**B) Peptide 1b in untreated LasR.** A Graphical fragment map correlating the relevant peptide sequence with the observed fragmentation ions of Peptide 1b. Left, the 5+ charge state (m/z 785.76758) corresponds to a peptide containing $Cys^{188}$-SOH modified by IAM (theoretical molecular mass, 3924.83249Da). Right, MS/MS fragmentation of the fragment (m/z 785.76758), in which the $y_2^+$ peak represents the last two amino acid residues (IR) of Peptide 1b and the $b_2^+$ peak represents the first four amino acid residues (EV) of Peptide 1b. Other peaks follow the same pattern. Since the MS/MS spectrogram is too long, it is separated into two (top and bottom) panels.

**A (Peptide 1c)**

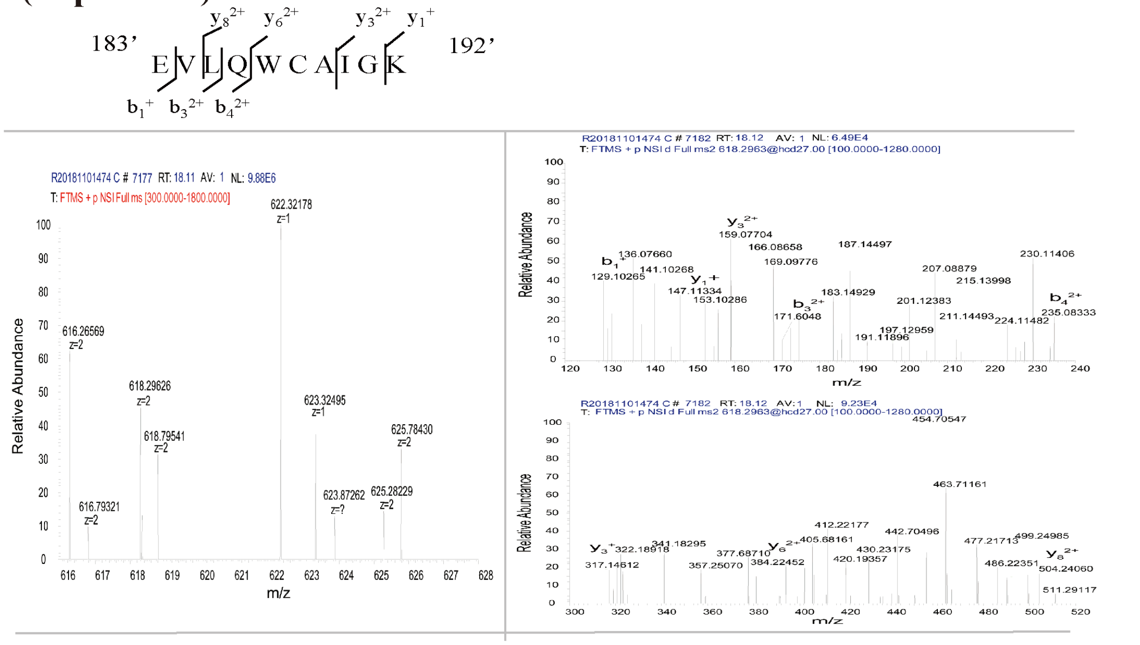


Sequence: EVLQWCAIGK, C6-SS-Carbamidomethyl (88.99300 Da) Charge: +2, Monoisotopic m/z: 618.29626 Da (-2.7 mmu/-4.37 ppm), MH+: 1235.58525 Da.

**B (Peptide 1d)**

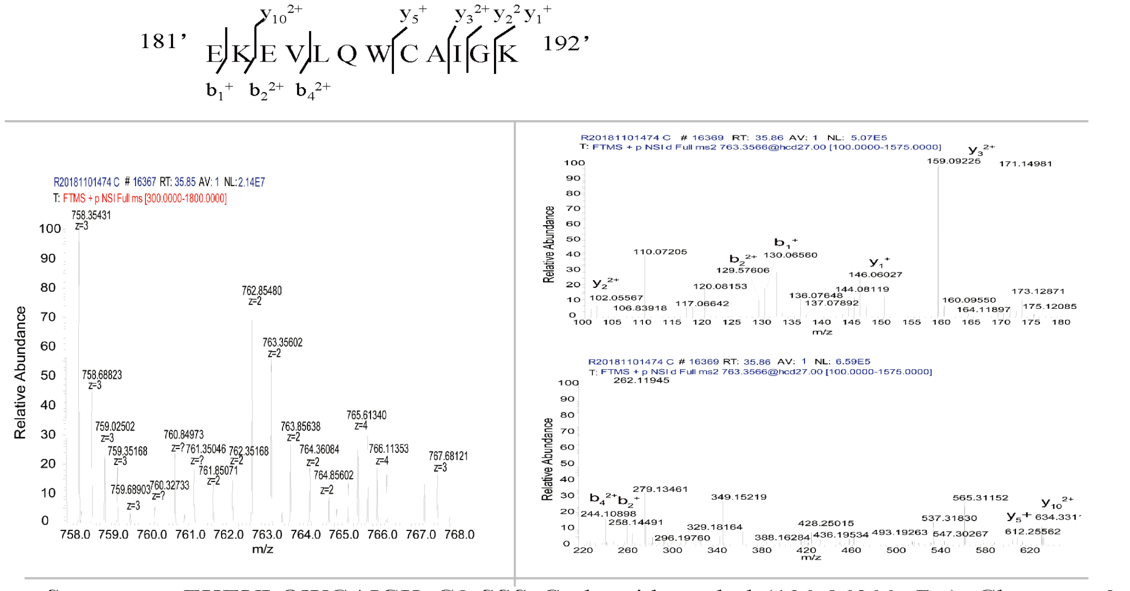


Sequence: EKEVLQWCAIGK, C8-SSS-Carbamidomethyl (120.96300 Da) Charge: +2, Monoisotopic m/z: 762.85480 Da (+2.05 mmu/+2.69 ppm), MH+: 1524.70232 Da.

**Figure S5. LTQ-Orbitrap tandem mass spectrometry analysis of LasR (Peptides 1c&1d).**

**A) Peptide 1c in $HS_n^-$-treated LasR.** A Graphical fragment map correlating the relevant peptide sequence with the observed fragmentation ions of Peptide 1c. Left, the 2+ charge state (m/z 618.29626) corresponds to Peptide 1c containing Cys[188]-SSH persulfidation

modified by IAM (theoretical molecular mass, 1235.59063Da). Right, MS/MS fragmentation of the fragment (m/z 618.29626), in which the $y_1^+$ peak represents the last amino acid residue (K) of Peptide 1c and the $b_1^+$ peak represents the first four amino acid residues (E) of Peptide 1c. Other peaks follow the same pattern. Since the MS/MS spectrogram is too long, it is separated into two (top and bottom) panels.

**B) Peptide 1d in $HS_n^-$-treated LasR.** A Graphical fragment map correlating the relevant peptide sequence with the observed fragmentation ions of Peptide 1d. Left, the 2+ charge state (m/z 762.85480) corresponds to Peptide 1d containing Cys$^{188}$-SSSH trisulfidation modified by IAM (theoretical molecular mass, 1524.69819Da). Right, MS/MS fragmentation of the fragment (m/z 762.85480), in which the $y_a^+$ peak represents the last amino acid residue (K) of Peptide 1d and the $b_1^+$ peak represents the first four amino acid residues (E) of Peptide 1d. Other peaks follow the same pattern. Since the MS/MS spectrogram is too long, it is separated into two (top and bottom) panels.

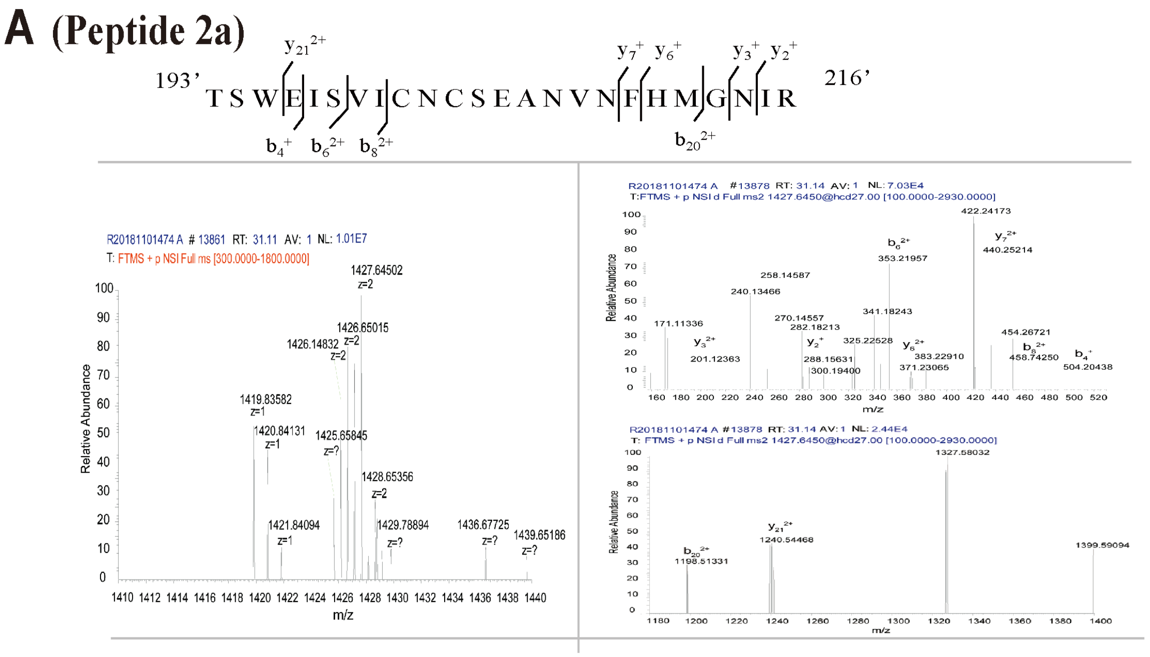


Sequence: TSWEISVICNCSEANVNFHMGNIR, C9-Carbamidomethyl (57.02146 Da), C11-Carbamidomethyl (57.02146 Da), M20-Oxidation (15.99492 Da) Charge: +2, Monoisotopic m/z: 1427.64502 Da (+6.05 mmu/+4.24 ppm), MH+: 2854.28276 Da.

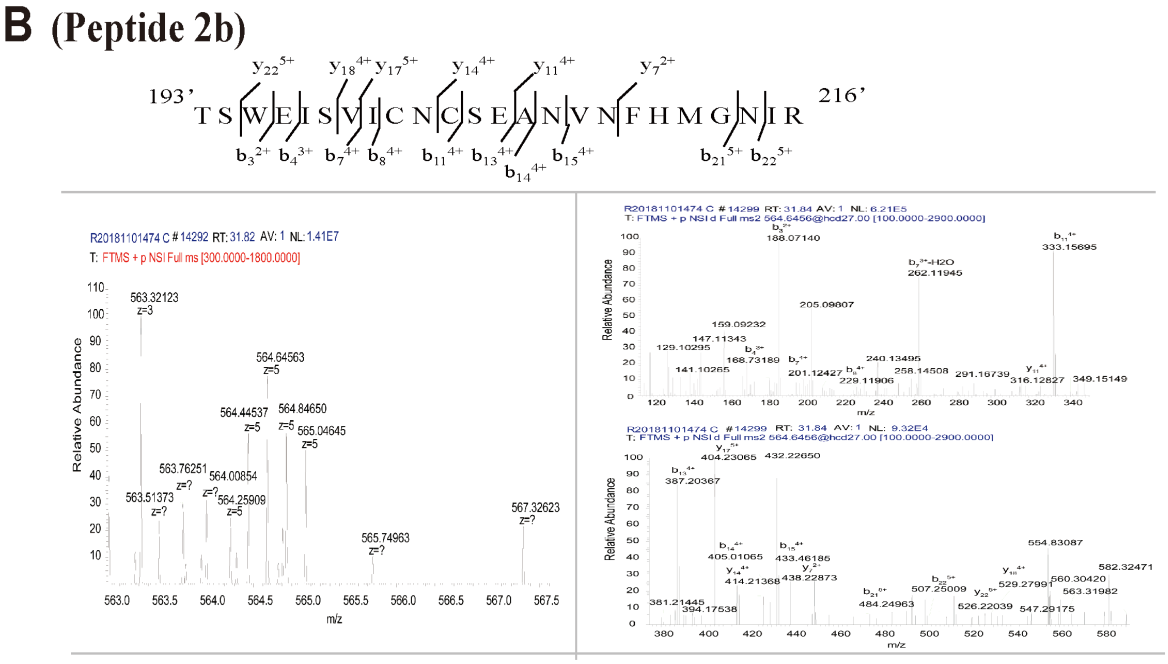


Sequence: TSWEISVICNCSEANVNFHMGNIR, C9-Sulfide (31.97207 Da), C11-tetrasulfide (62.00000 Da) Charge: +5, Monoisotopic m/z: 564.44537 Da (-1.43 mmu/-2.53 ppm), MH+: 2818.19776 Da.

**Figure S6. LTQ-Orbitrap tandem mass spectrometry analysis of LasR (Peptides 2a&2b).**

**A) Peptide 2a in DTT-treated LasR.** A Graphical fragment map correlating the relevant peptide sequence with the observed fragmentation ions of Peptide 2a in DTT-treated LasR. Left, the 2+ charge state (m/z 1427.64502) corresponds to Peptide 2a containing $Cys^{201}$ and

Cys$^{203}$ modified by iodoacetamide (IAM) (theoretical molecular mass, 2854.27058Da). Right, MS/MS fragmentation of the fragment (m/z 1427.64502), in which the $y_2^+$ peak represents the last two amino acid residues (IR) of Peptide 2a and the $b_4^+$ peak represents the first four amino acid residues (TSWE) of Peptide 2a. Other peaks follow the same pattern. Since the MS/MS spectrogram is too long, it is separated into two (top and bottom) panels.

**B) Peptide 2b in $HS_n^-$-treated LasR.** A Graphical fragment map correlating the relevant peptide sequence with the observed fragmentation ions of Peptide 2b. Left, the 5+ charge state (m/z 564.44537) corresponds to the pentasulfide-containing peptide of Peptide 2b without modification by IAM (theoretical molecular mass, 2818.20481Da). Right, MS/MS fragmentation of the fragment (m/z 564.44537), in which the $y_7^+$ peak represents the last amino acid residue (FHMGNIR) of Peptide 2b and the $b_3^+$ peak represents the first four amino acid residues (TSW) of Peptide 2b. Other peaks follow the same pattern. Since the MS/MS spectrogram is too long, it is separated into two (top and bottom) panels.

**Table S1. Strains and plasmids used in this study.**

| **Strain/plasmid** | **Characteristic** | **Source** |
|---|---|---|
| ***Escherichia coli* strains** | | |
| DH5α | Cloning strain | Invitrogen |
| BL21(DE3) | Cloning strain | Invitrogen |
| S17-1 | Transfer strain | Teng [a] |
| ***P.aeruginosa* strains** | | |
| PAO1 | Wild type | ATCC 15692 |
| PaΔ$H_2S$ | PAO1 mutant with *cbs*, *cse*, *mst* and *cysI* genes disru pted | This study |
| Pa3K | PAO1 mutant with *pdo* and *sqr* genes disrupted | This study |
| Pa7K | PAO1 mutant with *cbs*, *cse*, *mst, cysI*, *pdo* and *sqr* ge nes disrupted | This study |
| Δ*lasR* | PAO1 mutant with *lasR* gene disrupted | This study |
| ***Plasmids*** | | |
| pK18mobsacBtet | $Km^r$ and $Tet^r$, *sacB*, RP4 oriT, ColE1 ori; suicide vector | This study |
| Ptrc99a | Amp,broad host range | Our lab |
| pBBR1mcs2 | Kmr, broad host range | Kovach [b] |
| pBBR1mcs5 | Gm, broad host range | Kovach [b] |
| pBBR2-*Ppsqr* | pBBR1mcs2 containing *sqr from Pseudomonas putid a* S16 | Our lab |
| Ptrc-$P_{lacI}$-*lasR*-$P_{rhlR}$-*mkate* | The trc promotor of Ptrc99a was replaced by lac pro motor and with *lasR and rhlR operon* | This study |
| Ptrc-$P_{lacI}$-*lasR/C79S*-$P_{rhlR}$-*m kate* | Ptrc-$P_{lacI}$-*lasR*-$P_{rhlR}$-*mkate* with Cys79Ser | This study |
| Ptrc-$P_{lacI}$-*lasR/C188S*-$P_{rhlR}$-*mkate* | Ptrc-$P_{lacI}$-*lasR*-$P_{rhlR}$-*mkate* with Cys188Ser | This study |
| Ptrc-$P_{lacI}$-*lasR/C201S*-$P_{rhlR}$-*mkate* | Ptrc-$P_{lacI}$-*lasR*-$P_{rhlR}$-*mkate* with Cys201Ser | This study |
| Ptrc-$P_{lacI}$-*lasR/C203S*-$P_{rhlR}$-*mkate* | Ptr-$P_{lacI}$-*lasR*-$P_{rhlR}$-*mkate* Cys203Ser | This study |
| pET30a | Kmr, expression vector | Invitrogen |
| pET30-LasR | pET30a containing LasR with N terminal his-tag | This study |

[a] Teng F, Murray BE, Weinstock GM. 1998. Conjugal transfer of plasmid DNA from *Escherichia coli* to enterococci: a method to make insertion mutations. Plasmid 39:182–186. doi:10.1006/plas.1998.1336.

[b] Kovach ME, Elzer PH, Hill DS, Robertson GT, Farris MA, Roop RM 2nd, Peterson KM. 1995. Four new derivatives of the broad-host-range cloning vector pBBR1MCS, carrying different antibiotic-resistance cassettes. Gene. 166:175-176.

[c] An rbs sequence (aaagaggagaaa) was inserted before *mkate*.

## Table S2. Primers used in this study.

| Primers | Sequence (5'-3') | Usage |
|---|---|---|
| Cu11 | CTATGACATGATTACGAATTTGGCGGCAGCACCAGCCTACAG | Flanking up region for *cbs/cse* disruption |
| Cu12 | CGATCCTTGATGGAGCCGCCG | |
| Cd11 | GGCGGCTCCATCAAGGATCGGGGCATTGGTGACTCGCTGATCC | Flanking down region for *cbs/cse* disruption |
| Cd12 | CCGGGTACCGAGCTCGAATTCTGGTGGAACAGAGCGGCGTGC | |
| Yu11 | CTATGACATGATTACGAATTCGTGCTGACCAAGGTGCGC | Flanking up region for *cysI* disruption |
| Yu12 | TAGAGACCGTTCTGCAGGCGC | |
| Yd11 | CGCCTGCAGAACGGTCTCTACCGGACCGAGGAAGAACCC | Flanking down region for *cysI* disruption |
| Yd12 | CCGGGTACCGAGCTCGAATTCAGCTACATAGACTGGGTCGTGG | |
| Mu11 | CTATGACATGATTACGAATTGCCTTCACCGACGACCTCGAC | Flanking up region for *mst* disruption |
| Mu12 | CGGCAGTCGAGGACCAGCAG | |
| Md11 | CTGCTGGTCCTCGACTGCCGTCGCTTACTGCGGCTCCGGC | Flanking down region for *mst* disruption |
| Md12 | CCGGGTACCGAGCTCGAATTGACCACTTCCCGGCCGTCG | |
| lasR-del-1 | AGCTATGACATGATTACGAATTTCTGGGAGCTGGAGCATGG | Flanking up region for *lasR* disruption |
| lasR-del-2 | ACTGACCGTCGGGTCGAC | |
| lasR-del-3 | GCGGGTCGACCCGACGGTCAGTGCCAATGTGAACTTCCATATGG | Flanking down region for *lasR* disruption |
| lasR-del-4 | CCCCGGGTACCGAGCTCGAATTGAGTTCGATGCGCAAGGC | |
| his-lasR-1 | GTATGAAAGAAACCGCTGCTATGGCCTTGGTTGACGGTTTTCTTGA | LasR recombinant expression |
| his-lasR-2 | TCAGAGAGTAATAAGACCCAAATTAACGGC | |
| pET30a-1 | AGCAGCGGTTTCTTTCATACCA | |
| pET30a-2 | TTGGGTCTTATTACTCTCTGACACCACCACTGAGATCCGGC | |
| Ptrc-F | CGAAATTAGGACATCGTTGATGCGTTTCTACAAACTCTTTTTGTT | |
| Ptrc-R | AAAAGGCCATCCGTCAGGAT | |
| $P_{rhlR}$-mkate-1 | ATCCTGACGGATGGCCTTTTGTTTCATGGAATTGTCACAACCG | |
| $P_{rhlR}$-mkate-2 | TCAACGATGTCCTAATTTCGACGG | |
| lasR-1 | TCTCATCCGCCAAAACAGCCTCAGAGAGTAATAAGACCCAAATTAACGG | Primers for construction of Ptrc-$P_{lacI}$-*lasR*-$P_{rhlR}$-*mkate* |
| lasR-2 | TTTCACACAGGAAACAGACCATGGCCTTGGTTGACGGTT | |
| Ptrc-F1 | GGTCTGTTTCCTGTGTGAAATTG | |
| Ptrc-F2 | GGCTGTTTTGGCGGATGAGA | |
| Ptrc-lac-1 | GCAGTGATTTACGACCTGCAC | |
| Ptrc-lac-2 | GGCTCGTATGTTGTGTGGAATTTCACACAGGAAACAGACCATG | |
| lac-1 | TGCAGGTCGTAAATCACTGCTCATGCCGTTTGTGATGGCTTC | |
| lac-2 | AAATTCCACACAACATACGAGCCGG | |
| qPCR-lasB-F | ACCATGTTCTATCCGCTGGT | |
| qPCR-lasB-R | AGAACGCTTCGTTCATTCCG | |
| qPCR-lasI-F | AAGTTGCGTGCTCAAGTGTT | Primers for RT-qPCR |
| qPCR-lasI-R | ATCGAGAATTCGCCAGCAAC | |
| qPCR-rplS-F | ATACCGTGATCGTCCAGGTC | |
| qPCR-rplS-R | GGCTGTAGGTCTGGAAGGTA | |
| qPCR-rhlR-R | CTGGGCTTCGATTACTACGC | |
| qPCR-rhlR-R | CCCGTAGTTCTGCATCTGGT | |

**Table S3. List of genes significantly down regulated in PaΔH2S ( ≥5-fold) relative to the wild-type strain PAO1.**

| Gene_id | Gene name | $log_2$.Fold_ change | Description | [a]Regulated by LasR? |
|---|---|---|---|---|
| PA2853 | oprI | 5.4888 | outer membrane lipoprotein OprI | |
| PA3479 | rhlA | 5.3183 | rhamnosyltransferase subunit A | Yes |
| PA4306 | flp | 5.1465 | type IVb pilin Flp | |
| PA4224 | pchG | 5.0771 | pyochelin biosynthetic protein PchG | |
| PA4944 | hfq | 6.0236 | RNA-binding protein Hfq | |
| PA3477 | rhlR | 5.135 | transcriptional regulator RhlR | Yes |
| PA4224 | pchG | 5.0771 | pyochelin biosynthetic protein PchG | |
| PA3724 | lasB | 5.0053 | elastase LasB | Yes |
| PA1871 | lasA | 4.9773 | protease LasA | |
| PA4230 | pchB | 5.0255 | isochorismate-pyruvate lyase | |
| PA1431 | rsaL | 4.9585 | regulatory protein RsaL | Yes |
| PA3478 | rhlB | 4.8727 | rhamnosyltransferase subunit B | Yes |
| PA1432 | lasI | 4.4812 | acyl-homoserine-lactone synthase | Yes |
| PA2591 | vqsR | 3.3081 | transcriptional regulator | Yes |
| PA1003 | MvfR | 3.704 | transcriptional regulator MvfR | Yes |
| PA1092 | fliC | 4.4516 | B-type flagellin | |
| PA1095 | - | 4.2259 | B-type flagellar protein FliS | |
| PA4525 | pilA | 4.621 | type 4 fimbrial protein PilA | |
| PA1899 | phzA2 | 3.8406 | phenazine biosynthesis protein PhzA | |
| PA1900 | phzB2 | 3.7006 | phenazine biosynthesis protein PhzB | |
| PA0996 | pqsA | 3.1703 | anthranilate--CoA ligase | Yes |
| PA0997 | pqsB | 3.9034 | hypothetical protein | Yes |
| PA0998 | pqsC | 3.7062 | hypothetical protein | Yes |
| PA0999 | pqsD | 3.9007 | 3-oxoacyl-ACP synthase | Yes |
| PA1000 | pqsE | 3.6412 | thioesterase PqsE | Yes |
| PA1130 | rhlC | 3.03 | rhamnosyltransferase | |
| PA2426 | pvdS | 2.9271 | extracytoplasmic-function sigma-70 factor | Yes |
| PA2587 | pqsH | 2.2108 | 2-heptyl-3-hydroxy-4(1H)-quinolone synthase | Yes |
| PA1754 | cysB | 3.6132 | transcriptional regulator CysB | |
| PA5366 | pstB | 3.5758 | ABC transporter ATP-binding protein | |
| PA1246 | aprD | 3.811 | alkaline protease secretion ATP-binding protein AprD | Yes |
| PA1247 | aprE | 3.5344 | alkaline protease secretion protein AprE | Yes |
| PA1248 | aprF | 3.5018 | alkaline protease secretion protein AprF | Yes |
| PA3584 | glpD | 4.3362 | glycerol-3-phosphate dehydrogenase | |
| PA1584 | sdhB | 4.0662 | succinate dehydrogenase iron-sulfur subunit | |
| PA5521 | - | 3.6808 | short-chain dehydrogenase | |
| PA2302 | ambE | 2.5615 | protein AmbE | Yes |
| PA2303 | ambD | 2.5755 | protein AmbD | Yes |
| PA2304 | ambC | 2.6372 | protein AmbC | Yes |
| PA3326 | - | 4.4329 | ATP-dependent Clp protease proteolytic subunit | Yes |
| PA0426 | MexB | 2.6353 | multidrug resistance protein MexB | |
| PA0427 | OprM | 2.3323 | outer membrane protein OprM | |
| PA3384 | phnC | 2.6233 | phosphonate ABC transporter ATP-binding protein | Yes |
| PA0425 | MexA | 2.7473 | multidrug resistance protein MexA | |

[a] We identify the genes that are regulated by LasR protein based on a published paper (28).

**Table S4. List of genes significantly up regulated in Pa$\Delta H_2S$ ( ≥ 5-fold) relative to the wild-type strain PAO1.**

| Gene_id | Gene name | $log_2$. Fold_ change | Description |
|---|---|---|---|
| PA0324 | - | 7.2716 | ABC transporter permease |
| PA0205 | - | 7.1812 | ABC transporter permease |
| PA3609 | potC | 7.026 | polyamine ABC transporter permease PotC |
| PA2924 | hisQ | 6.7349 | histidine ABC transporter permease HisQ |
| PA0204 | - | 6.6281 | ABC transporter permease |
| PA4859 | - | 6.5656 | ABC transporter permease |
| PA4860 | - | 5.7844 | ABC transporter permease |
| PA5383 | - | 8.1357 | hypothetical protein |
| PA4099 | - | 7.9936 | hypothetical protein |
| PA0790 | - | 7.8997 | hypothetical protein |
| PA0166 | - | 7.6124 | transporter |
| PA2923 | hisJ | 5.7473 | histidine ABC transporter substrate-binding protein HisJ |
| PA5386 | cdhA | 7.7244 | 3-hydroxybutyryl-CoA dehydrogenase |
| PA2295 | - | 5.4593 | ABC transporter permease |
| PA1113 | - | 3.0211 | ABC transporter ATP-binding protein/permease |
| PA1386 | - | 3.1932 | ABC transporter ATP-binding protein |
| PA4064 | - | 3.1915 | ABC transporter ATP-binding protein |
| PA3394 | nosF | 6.3041 | copper ABC transporter ATP-binding protein |
| PA1635 | kdpC | 5.0389 | potassium-transporting ATPase subunit C |
| PA3039 | - | 5.0173 | transporter |
| PA2350 | - | 4.7686 | methionine ABC transporter ATP-binding protein |
| PA0220 | - | 5.5798 | amino acid APC family transporter |
| PA1908 | - | 3.7616 | major facilitator superfamily transporter |
| PA3000 | aroP1 | 2.7517 | aromatic amino acid transporter AroP |
| PA5512 | mifS | 4.1193 | sensor histidine kinase MifS |
| PA2356 | msuD | 6.7111 | methanesulfonate monooxygenase |
| PA1724 | pscK | 5.9492 | type III export protein PscK |
| PA1695 | pscP | 4.0634 | translocation protein in type III secretion |
| PA1971 | braZ | 5.9384 | branched-chain amino acid transport system 3 carrier protein |
| PA1146 | - | 5.671 | iron-containing alcohol dehydrogenase |
| PA1284 | - | 4.1207 | acyl-CoA dehydrogenase |
| PA3061 | pelD | 5.4548 | pellicle/biofilm biosynthesis protein PelD |
| PA1927 | metE | 3.4388 | 5-methyltetrahydropteroyltriglutamate--homocysteine methyltransferase |
| PA5294 | - | 4.7242 | multidrug efflux protein NorA |
| PA2493 | mexE | 4.0596 | resistance-nodulation-cell division (RND) multidrug efflux membrane fusion protein MexE |
| PA2494 | mexF | 3.5557 | resistance-nodulation-cell division (RND) multidrug efflux transporter MexF |
| PA4599 | mexC | 4.2725 | resistance-nodulation-cell division (RND) multidrug efflux membrane fusion protein MexC |
| PA4598 | mexD | 3.1978 | resistance-nodulation-cell division (RND) multidrug efflux transporter MexD |
| PA4597 | oprJ | 3.3622 | multidrug efflux outer membrane protein OprJ |

**Table S5. Mass data from LTQ-Orbitrap tandem mass spectrometry.**

| | Modification | Observed mass ($MH^+$) | Calculated mass ($MH^+$) |
|---|---|---|---|
| Peptide 1a | RS-CAM | 1203.62114 | 1203.61909 |
| Peptide 1b | RS(+O) | 3924.80878 | 3924.83249 |
| Peptide 1c | RS(+S-CAM) | 1235.58525 | 1235.59063 |
| Peptide 1d | RS(+S-S-CAM ) | 1524.70232 | 1524.69819 |
| Peptide 2a | RS-CAM | 2854.28276 | 2854.27058 |
| Peptide 2b | RS-SSS-SR' | 2818.19776 | 2818.20481 |

Peptide mass was calculated on the website:

http://db.systemsbiology.net:8080/proteomicsToolkit/FragIonServlet.html
